# Transcriptional and neuroprotective effects of hexokinase-2 inhibitors administered after stroke

**DOI:** 10.1186/s12974-025-03594-1

**Published:** 2025-10-28

**Authors:** Seok Joon Won, Gӧkhan Uruk, Nguyen Mai, Chia-Ling Tu, Devran Ogut, Sona Asatryan, Ebony Mocanu, Rachel Shon, Kyungsoo Kim, Khukheper Awakoaiye, Kajsa Arkelius, Wenhan Chang, Neel S. Singhal, Raymond A. Swanson

**Affiliations:** 1 Department of Neurology, University of California San Francisco and San Francisco Veterans Affairs Medical Center, 4150 Clement St., San Francisco, CA 94121 USA; 2 Department of Medicine, University of California San Francisco and San Francisco Veterans Affairs Medical Center, San Francisco, CA 94158 USA

**Keywords:** Hexokinase, Microglia, Ischemia, Brain, Spatial transcriptomics

## Abstract

**Background:**

The delayed inflammatory response induced by stroke can cause secondary injury to peri-infarct tissue. Microglia and other immune cells that mediate this injury require increased glycolytic flux for pro-inflammatory activation. These cells, unlike neurons, astrocytes, and most other cell types, utilize hexokinase-2 (HK2) rather than hexokinase-1 for glycolysis. Accordingly, HK2 inhibitors may selectively target immune cells to suppress post-stroke inflammation. Here we compared the effects of the non-selective hexokinase inhibitor 2-deoxyglucose to the HK2-selective inhibitors lonidamine and 3-bromopyruvate on gene expression changes and secondary injury after stroke.

**Methods:**

Stroke was induced in mouse motor cortex by photothrombosis, and the hexokinase inhibitors were administered intraperitoneally beginning three hours after stroke. Gene expression in microglia and neurons was evaluated in the adjacent and more distant peri-infarct cortex using in situ spatially defined cell type-specific whole transcriptomic profiling. Tissue injury was quantified using immunohistochemical methods, and mouse functional impairment was assessed over two weeks after stroke by the cylinder, rotating beam, and skilled reaching tasks.

**Results:**

Peri-infarct microglia exhibited an upregulation in pro-inflammatory gene expression, while neighboring neurons showed an upregulation in cell stress/death pathways. These changes were spatially dependent, being greatest adjacent to the infarct edge. The microglial gene expression changes correlated with both morphological activation and increased CD11b expression. The three hexokinase inhibitors had similar effects on gene expression, consistent with a shared mechanism of action. They suppressed pro-inflammatory gene upregulation in peri-infarct microglia, attenuated the cell stress responses in neighboring neurons, and had minimal effect on gene expression in uninjured cortex, with the HK2-selective inhibitors having greater anti-inflammatory effects than 2-deoxyglucose. The HK2-selective inhibitors were also more effective in suppressing microglial morphology changes and CD11b expression, and in suppressing oxidative stress and neurite loss in peri-infarct neurons. Mice treated with the HK2 inhibitor 3-bromopyruvate for four days after stroke showed long-term improvement in functional outcomes.

**Conclusions:**

These findings confirm an essential role for glycolysis in post-stroke, pro-inflammatory microglial activation. Selective HK2 inhibitors provide a clinically applicable approach for suppressing microglial activation and thereby improving outcomes after stroke.

**Supplementary Information:**

The online version contains supplementary material available at 10.1186/s12974-025-03594-1.

## Introduction

Focal ischemic stroke results from occlusion of an artery to the brain or spinal cord, usually by a blood clot, and causes infarction (pan-necrosis) in the vascular territory involved. The injury resulting from stroke can be mitigated by rapid clot lysis or mechanical removal, but only a small minority of stroke patients can be treated safely or effectively by these methods [1, 2]. In addition to the tissue infarction that occurs within hours after stroke, a delayed, secondary injury develops in the peri-infarct tissue. Inflammation is a major cause of this secondary injury, and since the post-stroke inflammatory response takes many hours to days to fully develop, anti-inflammatory intervention is recognized as a clinically feasible approach that could potentially benefit almost all stroke patients [3, 4].

The post-stroke inflammatory response is triggered by alarmins and other factors diffusing out from the infarct. It consists of an initial microglial activation followed by reactive astrocytosis and influx of macrophages and other immune cell types [3, 4]. This innate immune response is an evolutionarily conserved first line of defense against microbial infections, and as such it entails release of cytotoxic reactive oxygen species, proteases, and cytokines.

Pro-inflammatory microglial and macrophage activation requires a large increase in glycolytic flux [5, 6]. The first step of glycolysis (glucose → glucose-6-phosphate) is catalyzed by hexokinase, of which there are five mammalian isoforms [7, 8]. Hexokinase-1 is the isoform most abundantly expressed by most cell types in brain, including neurons, astrocytes, oligodendrocytes, and vascular cells, but microglia and other immune lineage cells differ in that they express predominately hexokinase-2 (HK2), and further upregulate HK2 expression when activated [9, 10]. It remains uncertain why immune cells require HK2-dependent glycolysis when activated [11], but this property nevertheless identifies HK2 as a target for suppressing post-stroke inflammation.

HK2-selective inhibitors have previously been shown to suppress the innate immune response and improve outcomes in mouse models of sepsis, arthritis, Alzheimer’s disease, and other disorders [9, 10, 12–16]. Selective HK2 inhibitors are also in clinical trials for other conditions and have an excellent safety profile [8, 17, 18] In contrast to pharmacological HK2 inhibition, genetic HK2 downregulation produces variable and conflicting effects, particularly when the HK2 ablation is complete [9, 15, 19]. This difference between effects of the pharmacological inhibitors and genetic downregulation may be attributable to loss of protein scaffolding or other functions of HK2, which are distinct from its glucose-phosphorylating function.

The present study aimed to evaluate effects of HK2 inhibition when initiated at a time point with clinical applicability, several hours after stroke. We compared the efficacies of two HK2-selective inhibitors, lonidamine (LND) and 3-bromopyruvate (3BP), and a non-selective hexokinase inhibitor 2-deoxyglucose (2DG) [20]. Spatially-defined and cell type-specific whole transcriptome profiles were acquired from microglia and neurons to determine the effects of injury and drug intervention on microglial activation, interactions between microglia and neurons, and potential off-target drug effects. Histological assessments performed in parallel were used to correlate microglial gene expression with microglial morphology changes after stroke and to determine HK2 inhibitor effects on secondary neuronal injury in the peri-infarct cortex. These studies were accompanied by a long-term survival study to evaluate effects on motor impairment and recovery after stroke.

## Methods and materials

### Animals

Studies were approved by the animal studies committees at the San Francisco Veterans Affairs Medical Center, and were performed in accordance with the National Institutes of Health Guide for the Care and Use of Laboratory Animals. Data were acquired and reported in accordance with the ARRIVE 2.0 guidelines [21]. C57BL/6 mice were obtained from the Jackson Laboratories. Each experiment included equal numbers of male and female mice, age 3–5 months old. There were no premature animal deaths during the study. Mice were arbitrarily assigned to the various treatment conditions and approximately equal ages in all groups. In each experiment the number of male and female mice was equal or, where odd number so mice were used, differed by one.

### Stroke induction

Mice were anesthetized with 2% isoflurane in 70% N_2_O/balance O_2_ delivered through a ventilated nose cone. Rectal temperature was maintained at 37.0 ± 0.5 °C with a homeothermic blanket throughout the surgical procedure. A photothrombotic infarct was induced by the Rose Bengal technique [22, 23]. For studies involving functional recovery, the infarct was placed on the motor cortex contralateral to the dominant paw, as assessed during acclimation to the skilled reaching task. After exposing the skull by skin incision, an isosceles right triangle-shaped adaptor (3 mm base x 3 mm height) was centered over the primary motor cortex (1.0 mm anterior, and 1.5 mm lateral to bregma) and connected to a white light source (KL 1500 LCD, SCHOTT North America Inc., Southbridge, MA) with a 2 mm diameter fiber optic cable. Rose Bengal (Sigma-Aldrich, St Louis, MO; 20 mg/kg) dissolved in saline was infused into the retro-orbital sinus for 30 s, and light was transmitted through the fiber optic cable for 15 min. Sham ischemia animals were injected with saline only but were otherwise treated identically. The incisions were sutured, bupivacaine (6 mg/kg) and buprenorphine SR (0.1 mg/kg) were administered subcutaneously, and the mouse was moved to a warm recovery chamber until awake and ambulatory. Vehicle solution (10% DMSO in saline), 2-deoxyglucose (2DG, 250 mg/kg), lonidamine (LND, 50 mg/kg) and 3-bromopyruvate (3BP, 5 mg/kg) were injected intraperitoneally (i.p.) at the indicated time points.

### Immunohistochemistry

Anesthetized mice were perfused with cold saline (0.9% NaCl) followed by a 4% solution of paraformaldehyde in phosphate-buffered saline, pH 7.4 (PFA). Brains were removed and post-fixed with PFA for 24 h, then immersed for another 24 h in 20% sucrose for cryoprotection. The brains were then frozen and 40 μm coronal sections were prepared with a cryostat. The fixed brain sections were incubated for 30 min in 10 mM sodium citrate (pH 6.0) at 80 °C for antigen retrieval, then pre-incubated in a blocking buffer (2% donkey serum, 0.3% Triton X-100 and 0.1% bovine serum albumin in 0.1 M phosphate buffer) at room temperature for 1 h and then incubated with the primary antibodies overnight at 4 °C. For CD11b immunohistochemistry, Triton X-100 and the antigen retrieval step were omitted. The antibody sources and dilutions used are listed in Supplemental Table 1. After washing, antibody binding was detected using fluorescent secondary antibodies listed in Supplemental Table 1. Stained sections were mounted on glass slides in a DAPI-containing anti-fade mounting medium (Vector laboratories, Burlingame, CA). For detection of cofilactin rods, the fixed sections were incubated with 100% methanol at −20 °C for 15 min and incubated with anti-cofilin-1 antibody in blocking buffer lacking detergent. Immunolabeling for NeuN was performed in a second step using standard, detergent-containing blocking buffer.

### Spatial gene expression

**Fig. 1:**
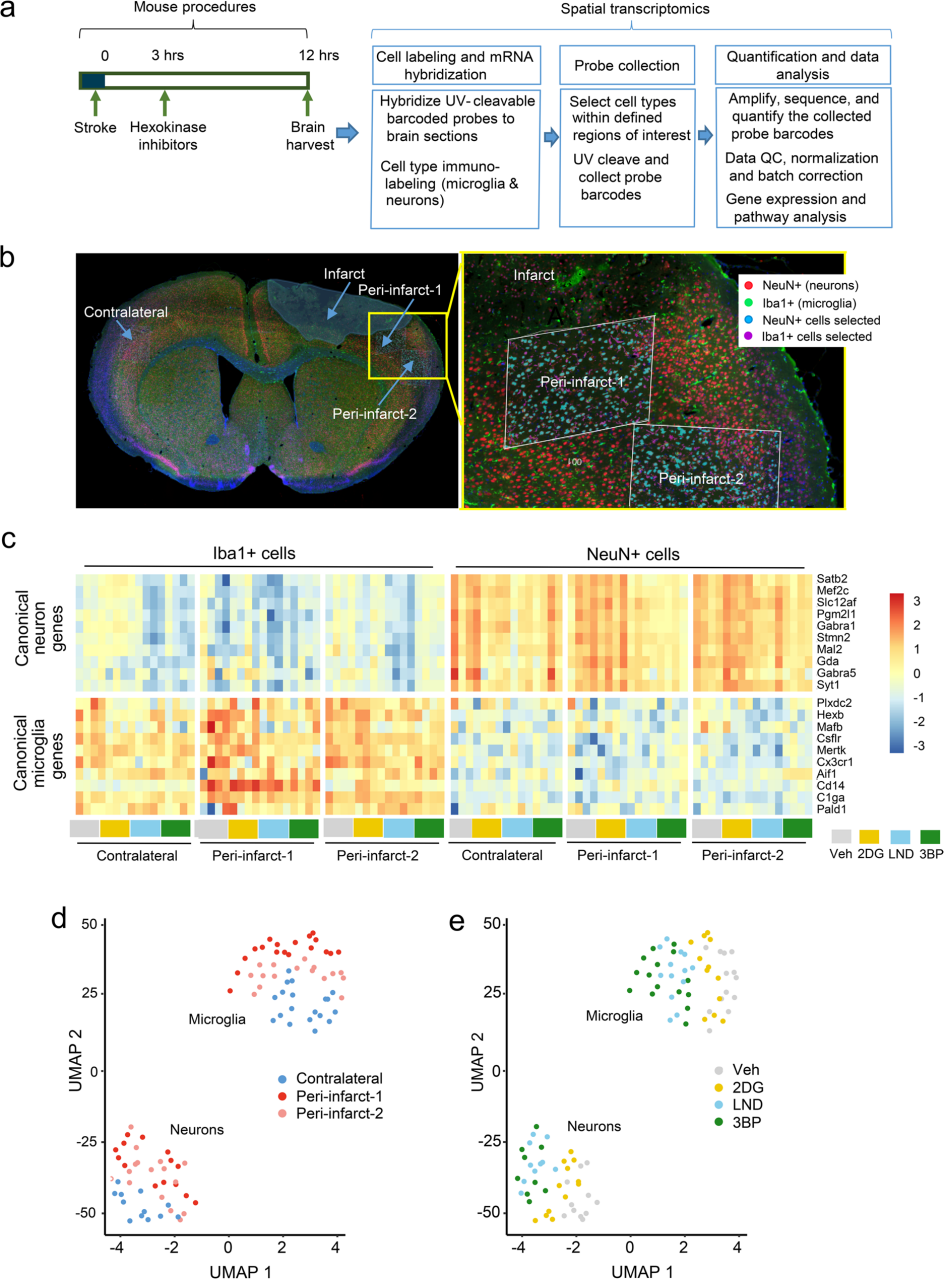
Spatial gene expression analysis of peri-infarct cortex.**a** Schematic of experimental workflow. **b** Immunolabeled brain section showing the spatial relationships between the infarct margin and the peri-infarct and contralateral probe collection areas. Microglia are identified by Iba1 immunoreactivity (green), neurons by NeuN (red) and nuclei by DAPI (blue). The yellow box in the low magnification view (left) shows location of the high magnification view (right). In the high magnification view, white parallelograms denote regions of interest in which the DNA barcodes were cleaved from the hybridization probes by UV light. Iba1-labeled cells selected for UV cleavage are displayed here in magenta. **c** Assessment of canonical neuronal and microglial gene expression in the NeuN + and Iba1 + cells corroborates neuronal and microglial labeling by these markers irrespective of sampling location or drug treatment group (2DG, 2-deoxyglucose; LND, lonidamine; 3BP, 3-bromopyruvate). (*n* = 4). **d** UMAP visualization of individual sample transcriptomic profiles from NeuN + and Iba1 + cells within the designated regions of interest. *n* = 4 mice under each of the 4 drug treatment conditions (*n* = 16 total). **e** The same UMAP data coded by drug treatment group

Paraffin-embedded brains were cut into 5-µm coronal slices, mounted on glass slides, and stored at −20 ^o^C with desiccant. The slides were subjected to deparaffinization, heat-induced antigen retrieval (10 min at 99 °C in Tris-EDTA pH 9.0), proteinase K digestion (0.3 mg/mL, 10 min at 37 °C), and fixation (5 min at room temperature in 10% NBF). Slides were then hybridized with a mouse Whole Transcriptome Atlas probe set (1:12 dilution, 16 h at 37 °C) and washed twice with a solution (2xSSC, 50% formamide). Microglia and neurons were identified by incubating with rabbit anti-Iba1 (FUJIFILM Waco) and guinea pig anti-NeuN (Synaptic System) at 4 °C overnight followed by AF488- conjugated anti-rabbit and AF647-conjugated anti-guinea pig secondary antibodies. Cell nuclei were visualized with 1 mM of SYTO83, a fluorescent DNA stain. Slides were loaded onto a GeoMx Digital Spatial Profiler (Bruker Spatial Biology) and imaged at 20X magnification, and 3 regions of interest (ROI) were selected per sample: (1) a parallelogram bordering the lateral edge of the infarct margin (as identified by loss of NeuN-positive neurons) and extending 500 μm laterally, termed here as “peri-infarct-1 cortex”; (2) a second parallelogram extending an additional 500 μm laterally, termed “peri-infarct-2 cortex”; and (3) the corresponding contralateral (uninjured) cortex, termed “contralateral cortex” (Fig. 1a, b). Within each ROI, photo-cleavable oligonucleotide bar codes conjugated to the gene-specific oligonucleotide probes [24] were liberated from the selected cells by UV (385 nm) illumination. The immunolabeled microglia and neurons were selected separately, and the cleaved oligonucleotide barcodes were collected by microcapillary aspiration into a 96-well plate. The oligonucleotide barcodes were then amplified by PCR using primers containing ROI-identifying sequences and Illumina adapter sequences. The resulting libraries were purified with AMPure XP beads, and then sequenced at 2 × 27 base pairs on a NovaSeq X Plus sequencer (Illumina).

### Histology image analysis

The regions of interest used for histology image analysis were defined in the same way as for spatial gene expression studies. Three coronal sections per mouse, spaced 240 μm apart, were used for each assessment. From each of the three sections, one image was analyzed from each of three regions of interest: peri-infarct-1, peri-infarct-2, and contralateral (Fig. 1b). Image quantification was performed by observers blinded to the mouse treatment conditions, as described [25]. For γH2Ax, the mean integrated fluorescence density was measured in the neuronal nuclei (as identified by NeuN immunolabeling) and expressed as the percent of neurons in which the signal intensity exceeded the 80th percentile value of neurons measured in homologous contralateral (uninjured) region [26]. Neurite length measurements were made after thresholding the NF-H images using the ImageJ Otsu function. The total NF-H neurite length in each image was quantified by summing the neurite lengths after skeletonizing the images in ImageJ and then normalized to the number of neuronal cell bodies per image as assessed by NeuN immunostaining [25]. For analysis of cofilactin rod formation, the images were thresholded using the Fiji Triangle function, and the area of cofilin-1 immunofluorescence was summed and normalized to the number of neurons per image. For CD11b, the mean integrated density of the fluorescence was measured after thresholding the images using the ImageJ Otsu function.

For Sholl analysis of microglial arborization [27], Z stacks of Iba1 immunostained images were compressed to form maximum intensity projections and thresholded using the “Make Binary” function in ImageJ. Analysis was conducted on individual cells using the Sholl plugin (ImageJ) with start radius = 0 pixels and step size = 1 pixel. Every fourth cell was analyzed in each image, excluding cells whose processes were cut off by the image edge. Data are displayed as the number of intersections formed by microglial processes at each radius from the cell center and the area under the curve (AUC) for each mouse. A separate analysis of total Iba1 process area per image (excluding the cell body area) was performed using the ImageJ particle size function.

### Infarct volume

Infarct volume was assessed in the 14 mice used for behavioral studies after euthanasia, at 21 days after ischemia. Twelve brain sections spaced 240 μm apart, spanning the entire infarct region, were immunostained with anti-NeuN and visualized by the diaminobenzidine method [28]. The area of neuronal loss in each section was calculated in Image J software, and the infarct volume in each brain was calculated summing these for each animal and multiplying by the 2.88 mm spanned by the 12 sections.

### Behavioral studies

Mice were evaluated with three tests to assess locomotor asymmetry and motor dexterity: the cylinder test, rotating beam test, and skilled reaching test. The mice were acclimated to handling over 2-weeks before stroke, during which time they were also acclimated to the rotating beam and the skilled reaching tasks. Post-stroke testing was performed on days 1, 4, 7, 10, 14, 15, and 17 days after stroke. All observers were blinded to the mouse treatment conditions. For the cylinder test, a mouse was placed in a beaker (12 cm height x 17 cm diameter) for 10 min while being video-recorded from above [29]. The beaker was washed with 10% bleach in between mice. The videos were reviewed to determine which forelimb touched the beaker surface during rearing behavior. Touches were counted when they occurred immediately after a mouse rearing, and no score was assigned if the mouse touched the beaker wall with both forepaws simultaneously. Results were expressed as the percentage of touches made by the impaired forelimb (contralateral to infarct) relative to the total touches recorded [(touches by contralateral forelimb)/(touches by either forelimb) x 100].

The Wishaw skilled reaching task was performed using a custom-made automated box as previously described for rats [30, 31], size-modified for use in mice. The mice were trained to reach through a 0.5 cm slit for a 14 mg rodent diet pellet (Bio-Serv, # F0071) before induction of stroke. Paw preference was noted during initial training sessions, and a reaching slit was placed to facilitate the use of the preferred paw. Mice were fasted overnight before the training sessions and fed 10% of their body weight after the training (minus the amount eaten during training). Mice were considered adequately trained when they could successfully grab and eat a pellet in over 30 of 60 consecutive trials. Approximately 60% of the mice achieved this metric and those that did not were excluded from the studies. Stroke was induced in the hemisphere contralateral to the dominant paw. During post-stroke testing, mice were given 60 opportunities to grab a pellet, and the percentage of successful grabs (pellets eaten) was scored.

The rotating beam test [32] requires mice to traverse the length of a rotating 145 cm long/1 cm diameter beam. The task is scored by the distance the mouse covers before falling off onto a safety net below. The mice were habituated to the rotating beam, with rotation speed increased from 0 rpm to 9 rpm. The mice completed 3 repetitions on the beam on each day of training. Mice displaying right paw preference were trained on a clockwise rotating beam and those with left paw preference on a counter-clockwise rotating beam. The beam was wiped clean with bleach between mice. Mice were considered adequately habituated when they could traverse the full 145 cm at a 9 rpm rotation speed on 3 consecutive trials. After stroke, the beam rotation speed was set at 9 rpm on days 1 and 4, at 15 rpm on days 7 and 10, and then at 20 rpm on days 14, 15, and 17. Each testing session consisted of 3 repetitions on the beam. The mean distance traveled was recorded as the score obtained for each testing day.

### Bioinformatics and statistics

Sequencing reads were aligned to mouse genome (MGSCv37) for production of raw counts. The raw gene counts were filtered for lowly expressed genes (< 30 CPM averaged across samples). We additionally filtered 214 genes known to be expressed in astrocyte processes [33–35], because the spatial transcriptomics method cannot exclude RNA from astrocyte processes traversing the neuronal and microglial nuclei of interest. These are listed in Supplemental Data File 13. The remaining gene counts were normalized with the variance stabilizing transformation algorithm, and differential gene expression was performed in R with DESeq2 [36]. Random forest classification and variable importance analysis for activated microglia gene signature generation were performed in R with nestedCV using nested cross-validation of the normalized microglial gene expression matrix of vehicle treated mice as the input [37]. Gene set variance analysis was performed using gene ontology (GO): Biological pathways annotations and the GSVA package [38]. Gene expression plots, heatmaps, and volcano plots were generated with ggplot2, pheatmap, and EnhancedVolcano, respectively [39–41]. CIBERSORTX analysis [42] was applied to the gene expression matrices to impute microglial cell fractions based on a signature matrix derived from the Human Microglial Atlas (HuMicA), a single cell RNA sequencing-based atlas classifying microglial expression patterns into 9 subtypes [43].

Experimental results were analyzed by unpaired t-test when only two groups were compared, or by ANOVA with Dunnett’s test for comparisons against a common reference group. For all figures, the “n” represents the number of mice evaluated.

## Results

We used the HK2- selective inhibitors lonidamine (LND) and 3-bromopyruvate (3BP) at doses previously shown to inhibit HK2 activity in mouse brain [9, 15]. These drugs were compared to the non-selective hexokinase inhibitor 2-deoxyglucose (2DG) used at a dose comparable to that previously used in studies of mouse brain ischemia [44, 45]. Under these conditions, 2DG induced mild sedation, while LND and 3BP had no discernible behavioral effect. The inhibitors were administered by intraperitoneal injection 3 h following stroke, and brains were harvested 9 h later for evaluation of microglial and neuronal gene expression by digital spatial profiling. Whole-transcriptome profiles were obtained from the peri-ischemic cortex abutting the infarct (denoted “peri-infarct-1”); from the adjacent region spanning 0.5–1.0 mm from the infarct edge (“peri-infarct-2”); and from the corresponding region of the contralateral uninjured cortex (“contralateral”; Fig. 1b). After filtering, quality control, and Q3 normalization [46] the transcriptomic profiles consisted of 19,740 genes (Supplemental Data Files 1 & 2). Profiles from the cells identified as neurons or microglia by immunolabeling showed the expected enriched expression of these cell-specific gene sets [47, 48] (Fig. 1c). Dimensionality reduction of the whole transcriptome data with uniform manifold approximation and projection (UMAP) confirmed the expected segregation of transcriptomes by cell type, while also revealing more modest patterns of segregation by region of interest and treatment condition (Fig. 1d, e). Although microglia and macrophages have different embryonic lineages, activated resident microglia and infiltrating macrophages have similar phenotypes and gene expression patterns [49]. For this reason and for brevity, we will not distinguish between these two cell types but instead refer to them collectively as “microglia” in the remainder of this work.

### HK2 inhibitors suppress pro-inflammatory gene expression in peri-infarct microglia

**Fig. 2:**
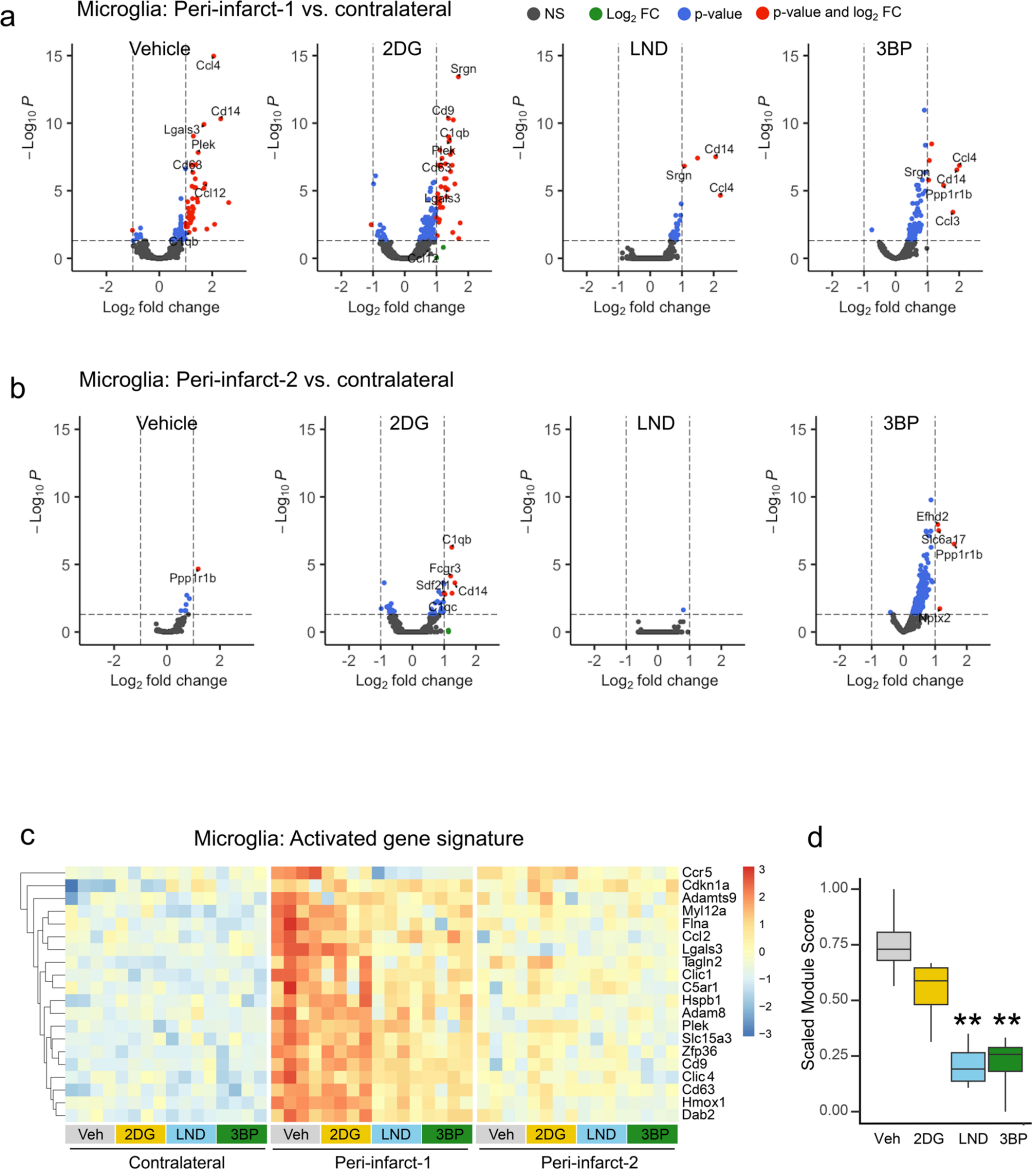
Effects of hexokinase inhibitors on peri-infarct microglial gene expression. Volcano plots show gene expression changes in peri-infarct-1 microglia (**a**) and peri-infarct-2 microglia (**b**) relative to microglia in the contralateral cortex under each drug treatment condition (2DG, 2-deoxyglucose; LND, lonidamine; 3BP, 3-bromopyruvate). Genes with log_2_ expression fold change (FC) > 1 and *p* < 0.05 are denoted by red dots; log_2_ FC > 1 and *p* > 0.05 by green dots; log_2_ FC < 1 and *p* < 0.05 denoted by blue dots, and not significant (NS) by black dots. **c **Heatmap showing relative expression of genes in the ischemia-activated gene set in each drug treatment condition and region of interest *n* = 4. **d** Box plot showing the normalized composite expression of the gene sets in peri-infarct-1 cortex. **p < 0.01 vs. vehicle by Dunnett’s test

In vehicle-treated mice, microglia in the peri-infarct-1 region showed a robust increase in pro-inflammatory gene expression relative to microglia in the contralateral cortex. Microglia in the more distant peri-infarct-2 region showed a smaller magnitude but otherwise similar pattern of gene upregulation (Fig. 2a, b). Effects of the HK2 inhibitors were assessed as comparisons between the peri-infarct vs. contralateral cortex under each drug treatment condition (Fig. 2a, b), and as comparisons between each of the HK2 inhibitors vs. vehicle in peri-infarct cortex (Suppl. Figure 1). By both methods the effects of 3BP and LND were comparable, consistent with a shared mechanism of drug action on the microglia. Full data sets for each of the microglial gene expression comparisons are provided as Supplemental Data Files 3–7.

A random forest classification and variable importance analysis of the gene expression profiles revealed an activated gene module most characteristic of peri-infarct-1 microglia. Genes in this group were found to include heme-oxygenase 1, the metalloproteinase genes ADAMTS9 and ADAM8, the chemokine receptor CCR5 and ligand CCL2, and others (Fig. 2c). The upregulation of this gene module in peri-infarct cortex was profoundly suppressed by the HK2 selective inhibitors LND or 3BP but to a lesser and non-significant extent by 2DG, as assessed with scaled module scores (Fig. 2d, Suppl. Figure 2). The activated gene module identified by the random forest classification showed individual gene and pathway overlap with a gene set previously identified as characteristic of disease-activated microglia (DAM) in neurodegenerative diseases [47, 50, 51]. Evaluation of the DAM gene set expression in the microglia showed it to be likewise upregulated in peri-infarct cortex and attenuated by the HK2 inhibitors (Suppl. Figure 3).

Deconvolution of microglial subtypes with CIBERSORTx analysis (Suppl. Figure 4) revealed significant differences among the differing regions of interest and drug treatment groups. The proportions of disease inflammatory macrophages (DIMs) and ribosomal disease associated microglia-2 (Ribo.DAM2) were increased and homeostatic-1 (Homeos1) microglia were decreased in the peri-infarct-1 cortex relative to control. Additionally, significant drug treatment effects were observed for DIMs (a microglia-like population derived from infiltrating monocytes) and for Homeostatic-3 (Homeos3) microglia (considered to be a non-activated microglial state) [43], with 3BP and LND increasing the relative abundance of both subtypes. No significant spatial × treatment interactions were identified.

**Fig. 3:**
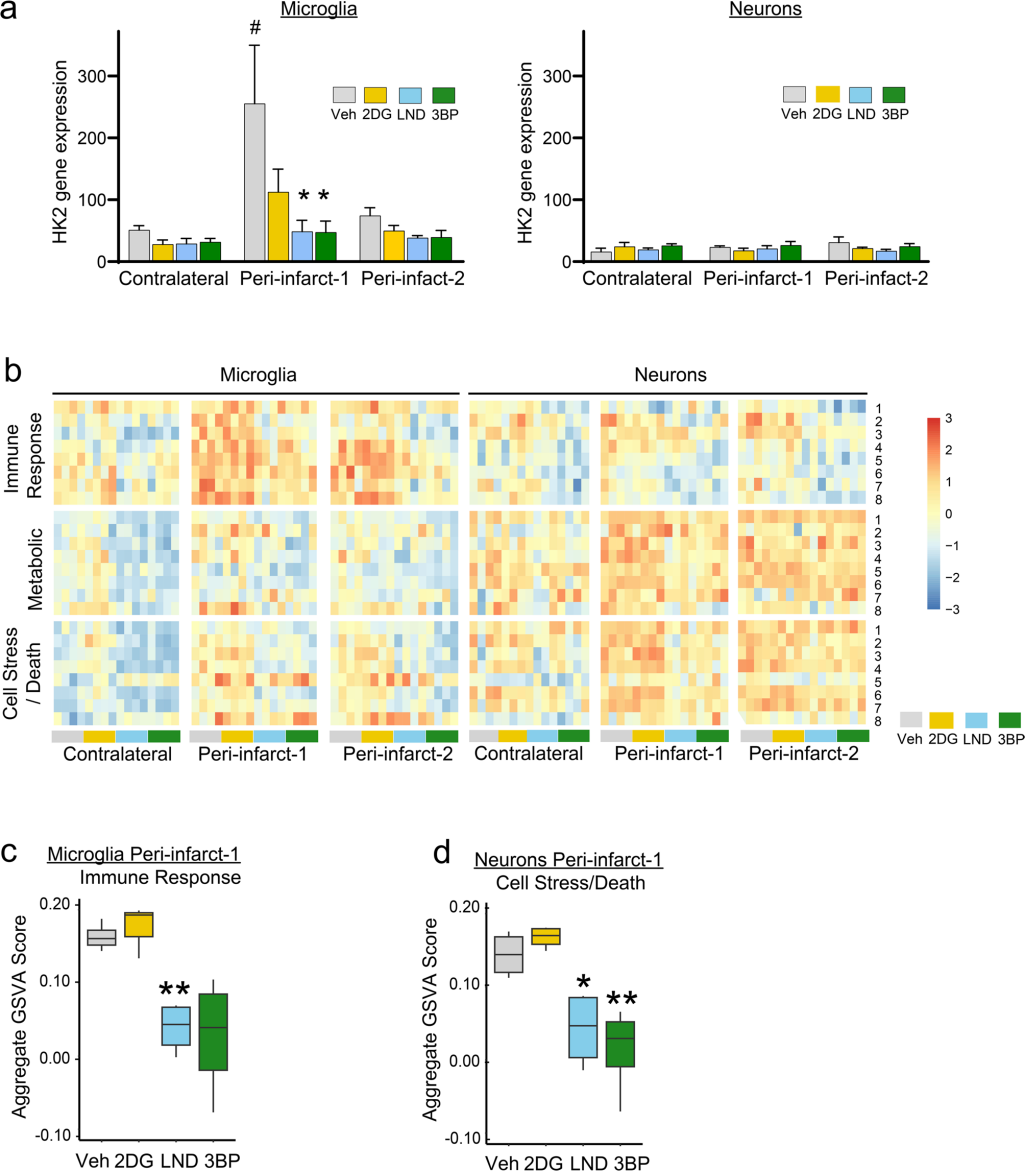
Microglial and neuronal HK2 expression and functional pathway analyses. **a **HK2 inhibitor effects on HK2 gene expression. n = 4. ^#^ *p* < 0.01 vs. contralateral; *p < 0.05 vs. vehicle. **b **Heatmap comparing z-scaled expression of the selected functional gene sets. The functional pathways within each pathway group are denoted by numbers on the right-hand side of the heatmap and listed in Supplemental Figure 6.**c, d **HK2 inhibitor effects on aggregated gene set variance analysis (GSVA) scores for the microglial immune response functional pathway and the neuronal cell stress/death functional pathway in peri-infarct-1 cortex. *p, 0.05, **p < 0.01 vs. vehicle

HK2 expression is reportedly higher at baseline in microglia and other myeloid cells than in neurons or other cell types and further increased by pro-inflammatory activation [10, 15, 19]. Our results agree with these reports, as we found that microglia in uninjured cortex exhibit a roughly 3-fold higher level of HK2 gene expression than neurons, with this increasing to 12-fold higher in peri-infarct-1 microglia (Fig. 3a). Remarkably, these increases in microglial HK2 expression were completely negated by 3BP or LND, and partially by 2DG. By contrast, the expression of HK2 in neurons was low at both basally and post-injury and unaffected by either proximity to the infarct margin or the HK2 inhibitors, supporting a microglia-specific action of the HK2 inhibitors.

### HK2 inhibitors indirectly affect neuronal gene

Although neuronal expression of the HK2 gene was not increased in peri-infarct cortex, the whole-transcriptome analysis revealed expression changes in many other neuronal genes (Suppl. Figure 5, Supplemental Data Files 8–12). The HK2-selective inhibitors attenuated the peri-infarct neuronal gene expression changes, while 2DG had less effect. Functional pathways affected by peri-infarct location and the HK2 inhibitors were evaluated by gene set variation analysis (GSVA) using Gene Ontology (GO): Biological Pathway (BP) annotations corresponding to inflammation, metabolism, and cell stress or death [38]. Peri-infarct microglia exhibited upregulation primarily in the immune response functional pathway, whereas neurons showed upregulation of cell stress/death pathways (Fig. 3b-d; Suppl Fig. 6). Of note, the changes in metabolic and cell stress/death neuronal pathways correlate more closely with the increased expression of immune responsive genes in peri-infarct-1 microglia than with changes in immune response gene expression in the neurons themselves. The HK2 inhibitors attenuated the changes in both cell types in the peri-infarct cortex, while having negligible effect on neuronal gene expression in the contralateral cortex (Suppl. Figure 5).

### HK2 inhibitors attenuate morphological markers of microglial activation

**Fig. 4:**
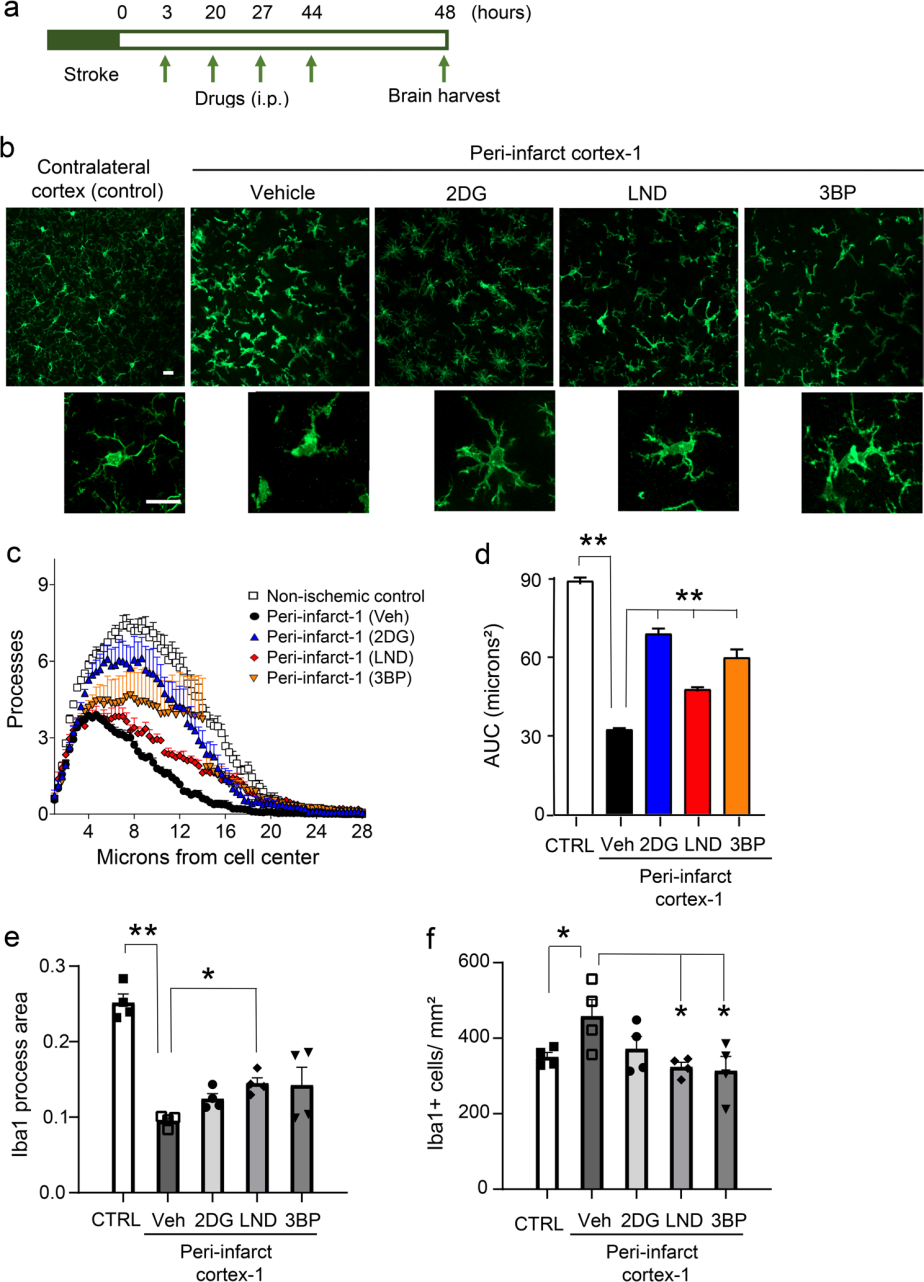
Effects of HK2 inhibitors on microglial morphology. **a** Experimental design. **b** Microglial morphology as shown by Iba1 immunolabeling 48 h after stroke. High-magnification images show cells with morphology representative of each treatment group. Scale bars = 10 μm. **c**,** d **Sholl analysis shows the mean number of microglial processes at distances from the cell center. These are quantified as area under the curve (AUC). **e **Mean microglial process area. **f** Microglia density. *n* = 4; *p < 0.05, **p < 0.01 by ANOVA and Dunnett’s test

**Fig. 5:**
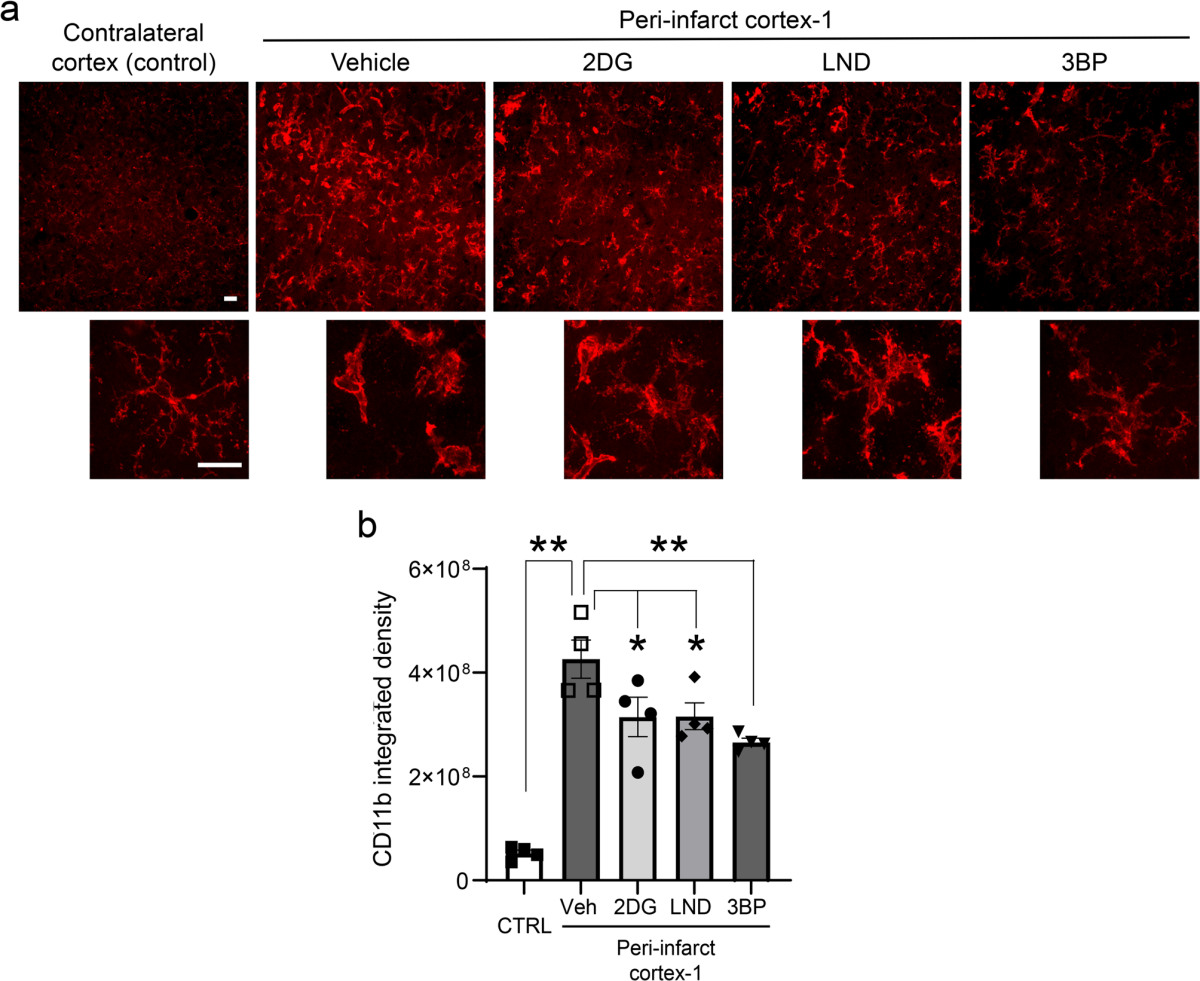
Effects of HK2 inhibitors on microglial CD11b expression. **a** Microglial CD11b immunoreactivity at 48 h after stroke. High-magnification images show cells with morphology representative of each treatment group. Experimental design as in Fig. 4. Scale bar = 10 μm. **b** Integrated density of CD11b immunoreactivity. *n* = 4; *p < 0.05, **p < 0.01 by ANOVA and Dunnett’s test

Assessments of microglial morphology were performed 48 h after stroke, at a time when this response is near maximal. Microglia in the peri-infarct-1 cortex exhibited the typical features of pro-inflammatory microglial activation: enlargement of the cell soma and retraction of cell processes (Fig. 4). These changes were accompanied by an increase in CD11b immunoreactivity (Fig. 5), which like the morphology changes is considered a hallmark of pro-inflammatory microglial activation [52, 53]. Both the pro-inflammatory morphology changes and the CD11b upregulation were suppressed by the HK2 inhibitors (Figs. 4 and 5). Microglia in the peri-infarct-2 region displayed a similar but less marked pattern of morphology changes in response to the stroke and drug treatments (Suppl. Figure 7).

### HK2 inhibitors attenuate neuronal injury in peri-infarct cortex

**Fig. 6:**
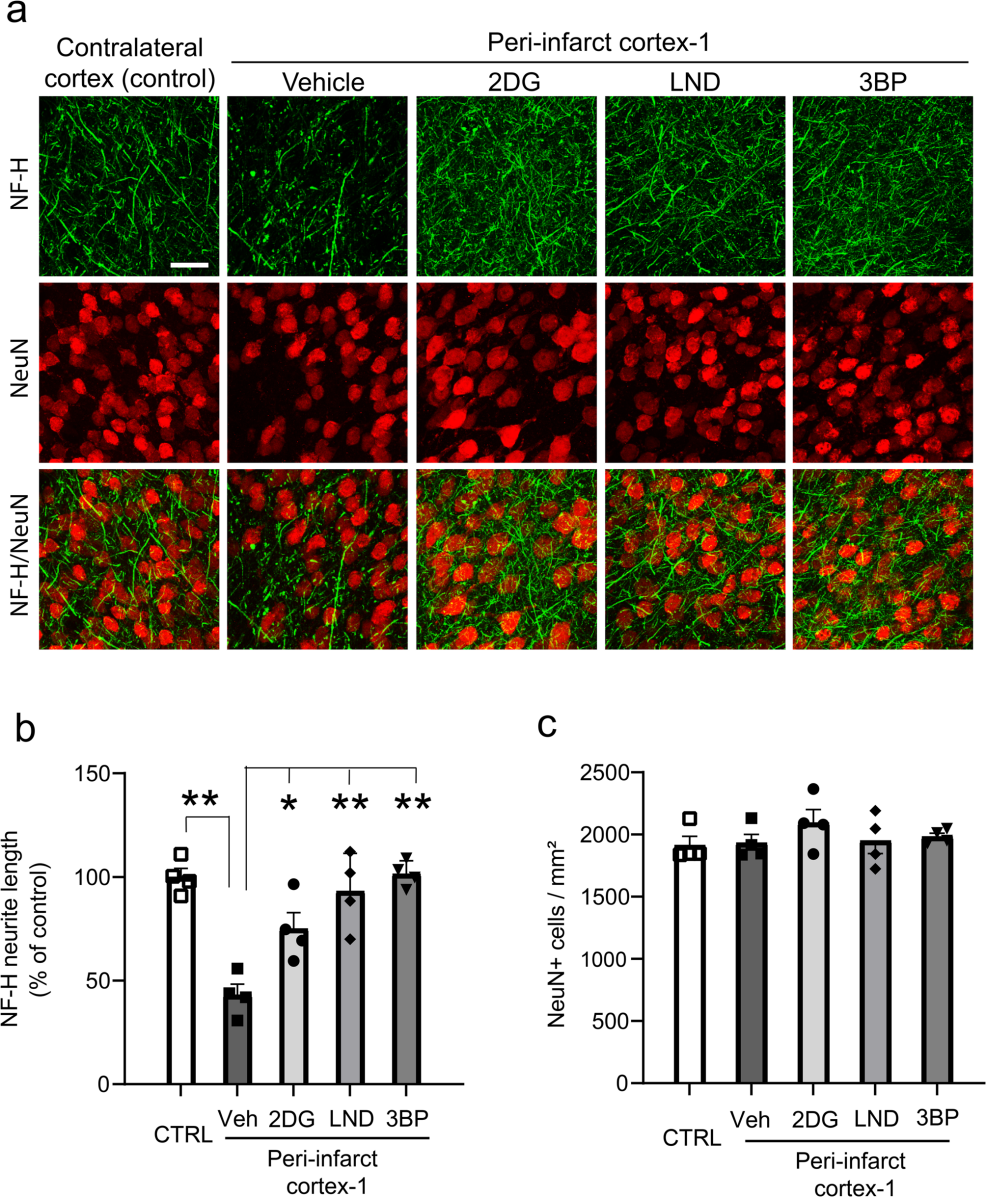
HK2 inhibitors administered after stroke reduce peri-infarct neurite loss. **a** Neurites are identified by immunostaining for neurofilament-H (NF-H, green), and neuronal soma by NeuN (red) 48 h after stroke. **b **Neurite density expressed as total NF-H process length/neuron and normalized to the control (contralateral) cortex. **c** There was no detectable loss of neuronal cell bodies in the peri-infarct cortex-1. *n* = 4); ^*^p < 0.05, **p < 0.01 vs. vehicle by ANOVA and Dunnett’s test

**Fig. 7:**
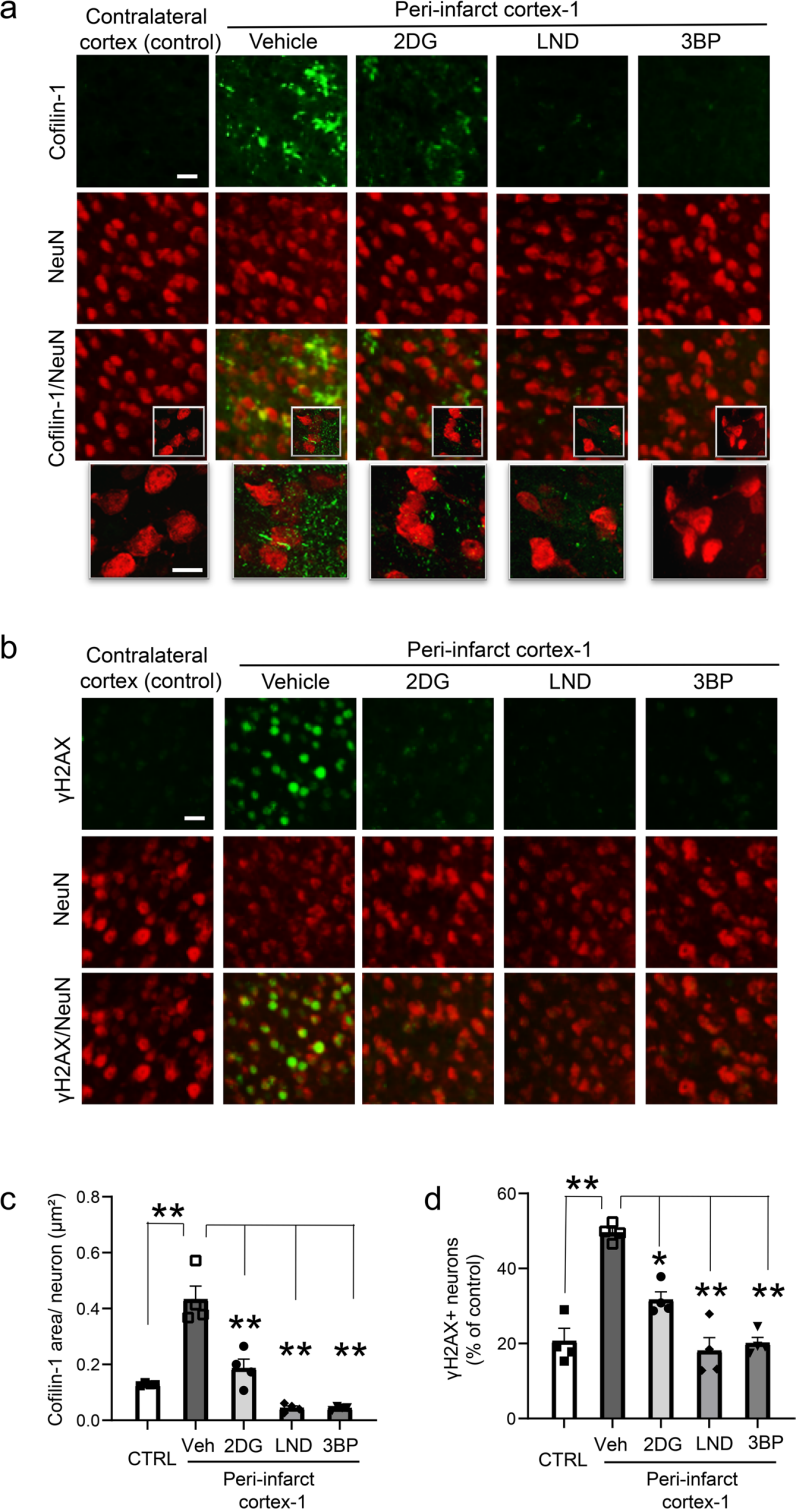
HK2 inhibitors suppress cofilactin rod formation and neuronal DNA damage in peri-infarct cortex. **a** Formation of cofilactin rods (cofilin-1, green) in peri-infarct-1 cortex 6 h after stroke. High magnification views show rod-like morphology of the cofilin-1 aggregates. Scale bars = 10 μm. **b** DNA damage as identified by foci of γH2Ax formation (green) in neurons (NeuN, red) of peri-infarct-1 cortex at 6 h after stroke. **c** Quantification of cofilactin rod formation. **d** Quantification of γH2Ax formation, expressed as number of cells with γH2Ax signal higher than the 80th percentile of neurons in the control (contralateral) cortex. *n* = 4, *p < 0.05; **p < 0.01 vs. vehicle by ANOVA and Dunnett’s test

Effects of HK2 inhibitors on secondary neuronal injury were also examined at the 48-hour time point. There was no detectable loss of neuronal cell bodies in peri-infarct cortex, but there was a more than 50% reduction in neurite density (Fig. 6). This reduction is consistent with the particular sensitivity of axons and dendrites to inflammation-induced injury [23, 25] The neurite loss was attenuated by the HK2 inhibitors, particularly LND and 3BP (Fig. 6). Inflammation-induced neurite loss results in part from the formation of cofilactin rods in response to local oxidative stress [25]. Here, cofilactin rod formation and oxidative stress were evident as early as 6 h after stroke (Fig. 7), with cofilactin rods identified as rod-like accumulations of cofilin-1, and oxidative stress indicated by nuclear DNA damage. All three HK2 inhibitors suppressed both neuronal oxidative stress and cofilactin rod formation, with LND and 3BP having larger effect sizes (Fig. 7).

**Fig. 8:**
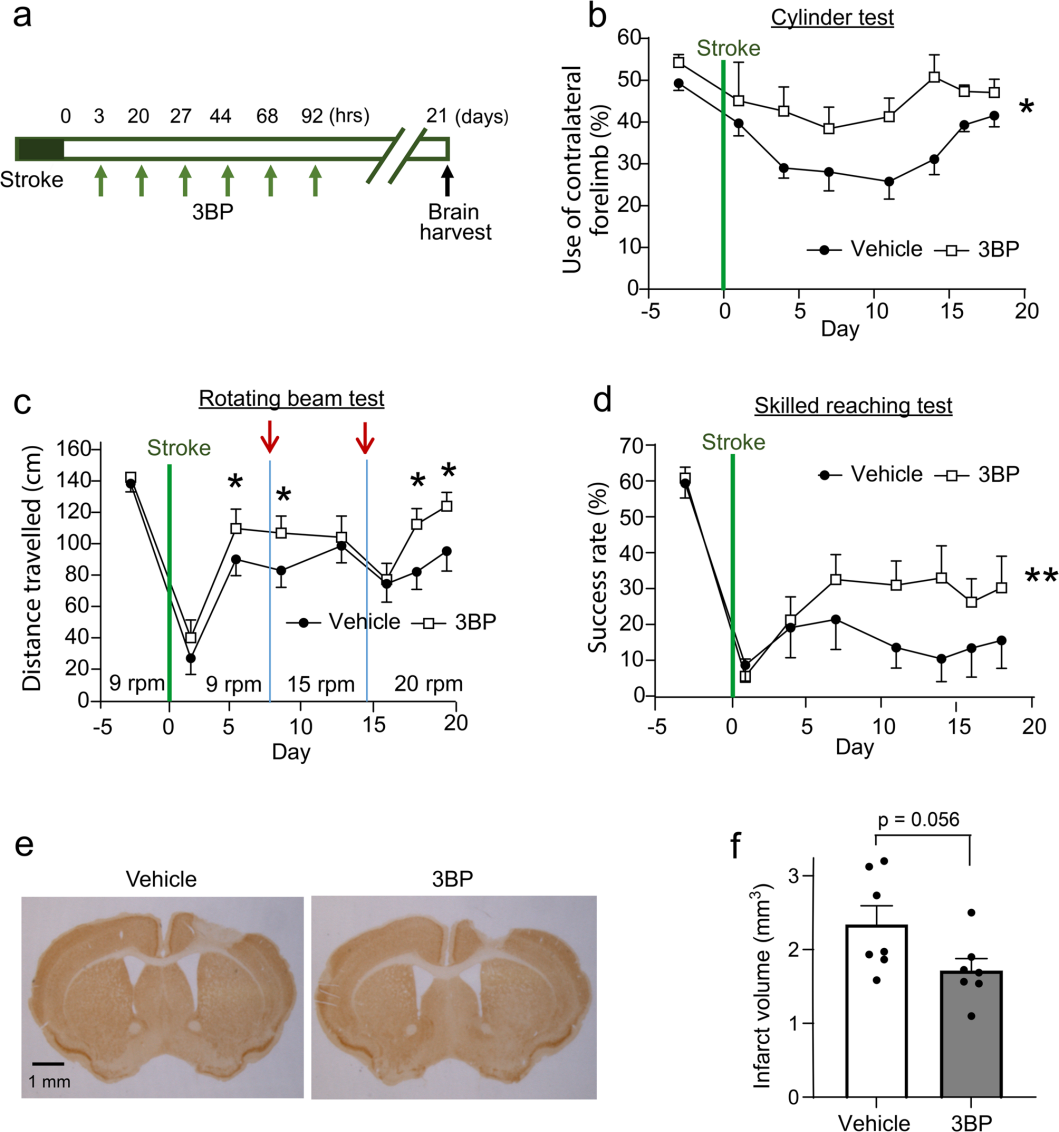
HK2 inhibitor administered after stroke reduce motor deficits and infarct size. **a** Experimental design. **b **Cylinder test. *p < 0.05 by repeated measures ANOVA over time. **c** Rotating beam test. Arrows denote days on which beam rotation speed was increased. *p < 0.05 by Student’s t-test on the designated days after speed increase. **d** Skilled reaching test. **p < 0.05 by repeated measures ANOVA over time (*n* = 7). **e** Coronal sections of mouse brain immunostained for NeuN to identify the infarct. **f** Brains harvested 21 days after stroke. Quantified infarct volumes in mice treated with 3BP or vehicle only following stroke. *p* = 0.05, *n* = 7

The effects of HK2 inhibitors on functional recovery were evaluated in mice treated with 3BP (or vehicle) initiated 3 h after stroke, with dosing repeated twice daily for the subsequent 3 days (Fig. 8 a). The mice were serially assessed the cylinder test, the rotating beam test, and the skilled reaching test over 21 days. The cylinder test showed increasing motor asymmetry in the initial days after stroke in both groups followed by gradual recovery, with the 3BP-treated group having overall better (less asymmetric) performance. The rotating beam test, in which the rotation speed is periodically increased, revealed superior performance in the 3BP-treated mice after each of these increases. The skilled reaching task similarly showed superior performance in the 3BP-treated group sustained over the entire 21-day testing interval (Fig. 8 b-d). Brains were harvested for measurements of infarct size at the end of the 21-day testing interval, and infarct size was found to be slightly smaller in the 3BP-treated mice (Fig. 8 e, f).

## Discussion

Our findings show that HK2 inhibitors can suppress the pro-inflammatory transcriptional response in peri-infarct microglia and reduce the cell stress/death transcriptional response in neighboring neurons. These transcriptional changes were associated with reduced peri-infarct microglial activation, reduced neuronal oxidative stress and neurite loss, and improved functional outcome. Given that the HK2 inhibitors had negligible effect on neuronal gene expression in uninjured cortex; that microglial HK2 expression is high relative to neuronal expression and further increased in peri-infarct cortex, and that activated microglia release cytotoxic metalloproteinases, cytokines and reactive oxygen species, we propose that the effects of HK2 inhibitors on neuronal oxidative stress, neurite loss, and gene expression in the peri-infarct cortex was indirect and secondary to the anti-inflammatory actions of these agents on neighboring microglia.

3BP, LND and 2DG all inhibit HK2 catalytic activity, but 2DG also inhibits HK1, and 3BP and lonidamine can have other off-target effects at higher doses [16, 54, 55]. It was thus of interest to compare the effects of these agents to one another. 3BP and LND had nearly identical effects on microglial gene expression, both in both pattern and magnitude, and likewise had nearly identical effects on microglial morphology and neuronal injury. These findings are consistent with a shared mechanism of action. Additionally, the much smaller effects of these agents on the microglial and neuronal transcriptome in the un-injured cortex, where HK2 expression is not upregulated, suggests an absence of significant off-target effects. The non-selective HK inhibitor 2DG had a somewhat different pattern of effects on peri-infarct microglial and neuronal gene expression, and had smaller effects on microglial morphology changes, neuronal oxidative stress. and neurite damage. The smaller magnitude of the 2DG effects may reflect a lesser degree of HK2 inhibition at the dose administered. The 2DG dose was limited by mouse sedation, likely due to neuronal HK1 inhibition and resultant suppression of neuronal glycolysis [56, 57].

The spatial transcriptomic approach permits simultaneous evaluation of gene expression in microglia and neighboring neurons. The functional pathway analyses showed that the pro-inflammatory microglial changes were associated with upregulation of neuronal stress/death pathways in both the peri-infarct-1 and peri-infarct-2 regions and that these changes in both microglia and neurons were lessened by the HK2-selective inhibitors. Given the near-absence of the HK2-selective inhibitors on neurons in undamaged cortex, together with the negligible HK2 gene expression detected in neurons, these results suggest that the HK2 inhibitor effects on neurons were indirect and secondary to their effects on microglia. The reduced neuronal oxidative stress in the HK2 inhibitor - treated peri-infarct cortex provides a potential mechanism for this interaction, given that activated microglia generate superoxide, nitric oxide, hypochlorous acid, and other and other reactive oxygen species [58] and HK2 has low expression in astrocytes and other brain cells [9, 10]. Nevertheless our data do not exclude the possibility that the inhibitors were additionally or alternatively acting on cell types other than microglia/macrophages.

We used a random forest classification to identify the gene set most characteristic of peri-infarct microglia compared to contralateral microglia. The genes and functional pathways in this set were found to overlap with the DAM gene set described in microglia associated with Alzheimer’s disease pathology [47], which exhibit innate immune activation and an increase in glycolytic metabolism. The upregulation of the DAM gene set in peri-infarct-1 microglia and suppression of this upregulation by HK2 inhibitors further suggests shared inflammatory and metabolic pathways between these microglial states [5, 59, 60].

The in situ transcriptomics approach enabled a simultaneous evaluation of transcriptional and morphological changes in microglia. In the peri-infarct-1 region, HK2 inhibitors attenuated the upregulation of pro-inflammatory gene expression observed in microglia at 12 h after stroke, and this correlated with a suppression of CD11b upregulation and the classical morphological changes observed at 48 h after stroke. Gene expression changes in the more distant peri-infarct-2 microglia were smaller in magnitude, and the changes in microglial morphology were likewise less pronounced. These observations confirm a correlation between the gene expression and pro-inflammatory morphological changes, but with the caveat that these gene expression assessments were intentionally performed at a time point preceding the histological evaluations, assuming some time lag between the two. Moreover, it is uncertain what transcriptional changes drive microglial morphology changes, or even if these are transcriptionally regulated.

The neurites (axons and dendrites) by which neurons communicate with one another have recently been shown to be particularly vulnerable to inflammation-induced injury through a process involving oxidative stress and formation of intra-neurite cofilin-1/actin aggregates (cofilactin rods) [25]. Our results here confirm oxidative stress, cofilactin rod formation, and neurite loss in peri-infarct cortex, all of which were attenuated by the HK2 inhibitors. The infarct size was also slightly smaller in the 3BP-treated mice, despite the 3 - hour interval between stroke and first drug administration, a result similar to that reported using delayed administration of fingolimod after permanent ischemia [61]. These findings, along with the attenuated upregulation of cytokine and protease genes in the mice treated with the HK2 inhibitors, suggest plausible mechanisms by which functional outcome was improved by 3BP, but do not exclude other possibilities.

The in situ transcriptomics approach employed here eliminates artifacts induced by gene expression changes during tissue dissociation and sorting during standard cell isolation methods [62] and allowed precise exclusion of microglia that are either remote from the infarct or had migrated into the infarct itself and thus unlikely to affect peri-infarct neuronal injury. Prior studies of microglial gene expression after stroke have used a variety of stroke models, tissue sampling areas, and methods for assessing gene expression [63–74]. These methodological differences complicate comparisons between studies, but our results are in general accord with the previous reports identifying upregulation of cytokine, chemokine, and protease-encoding genes in microglia. Prior studies using spatial transcriptomics in mouse models of stroke also highlight spatial heterogeneity in microglial responses [70–72, 74, 75].

However, a limitation of the DSP spatial transcriptomics approach is that it does not yield single-cell–resolved transcriptomic profiles, which could provide more granular data and transcriptomic profiles of additional cell types such as astrocytes, oligodendrocytes, and endothelia. Though only microglia and invading immune cells express high levels of HK2 relative to HK1, it is nevertheless possible that other cell types are affected, either directly or indirectly, by the HK2 inhibitors. Methods enabling spatial transcriptomics with true single-cell resolution are now emerging, and may be informative in future work to dissect the full cellular landscape of HK2 inhibitor effects on peri-infarct brain. Longitudinal studies incorporating additional, later time points will also be required to fully characterize the trajectory of HK2-related responses across the post-ischemic period.

An additional limitation to these studies is the use of contralateral, uninjured cortex as control rather than cortex from uninjured mice. This approach permits each mouse to serve as its own control; however, and even though the photothrombotic stroke model requires no craniotomy or neck incision, it remains possible that the stroke on one hemisphere may have had effects on gene expression or histological markers on the contralateral side.

Unexpectedly, the astrocyte-specific gene GFAP was among the genes robustly upregulated in both microglia and neurons of peri-infarct in the unfiltered transcriptome data set. A similar result was reported by [76] also using a spatial transcriptomic approach. GFAP is one of several genes that are highly upregulated in reactive astrocytes [77, 78]. Given that GFAP mRNA is transported through distal astrocyte processes [33–35], we suggest that the GFAP signal identified in peri-infarct microglia and neurons arises from mRNA in astrocyte processes traversing microglial and neuronal somas identified for probe photocleavage. This idea is supported by the increased detection, in both peri-infarct-1 neurons and microglia. of other mRNA transcripts that are found in astrocyte processes together with the lack of any increase in the nucleus-specific astrocyte transcripts SOX9, NFIA or S100B [33]. We therefore excluded the genes known to be expressed in astrocyte processes from the subsequent analysis of the microglial and neuronal transcriptome data [33] (Supplemental Data File 13).

Our findings contribute to the existing literature on glycolytic inhibition and the innate immune response. Ketogenic diet, which limits glucose flux through glycolysis, can suppress the innate immune response in a variety of settings, including stroke [79–81]. It was shown in the 1950 s that injury-induced inflammation can be robustly suppressed by 2DG [82], and 2DG was subsequently found to have anti-inflammatory effects in a variety of conditions including stroke [45, 83, 84]. 2DG is not a feasible therapeutic after stroke because the resulting suppression of HK1 activity in neurons and other cell types could exacerbate energy compromise in damaged or marginally perfused tissue. Salutary effects of selective HK2 inhibition have been reported in stroke [10, 16], though not using the clinically relevant, delayed administration or with the long-term behavioral endpoints assessments used here.

Several mechanisms have been identified by which glycolytic inhibition may affect the innate immune response. A reduced glycolytic flux reduces cytosolic NADH levels, which in turn suppresses the action of the master inflammatory transcription factor NFκB by promoting dimerization (inactivation) of the NADH –sensitive co-repressor CtBP [85]. In addition, non-transcriptional effects may be mediated by reduced capacity to generate the reactive oxygen species superoxide and nitric oxide, both of which need glucose as an obligate precursor [86–92]. Recent reports also identify additional, non-glycolytic actions specifically of HK2 that can influence immune responses. HK2 untethered from mitochondria can promote inflammasome assembly and thus IL1β production [93]. Untethered HK2 can also phosphorylate IκBα to cause IκBα proteolysis and resultant nuclear translocation of NFκB [94], but the effect of Hk2 inhibitors on these processes is not known. Our results demonstrated that the upregulation of HK2 gene expression in peri-infarct microglia was suppressed by HK2 inhibitors, suggesting that ongoing HK2 activity was required for maintaining the elevated HK2 expression. The interruption of this feed-forward loop may explain why HK2 inhibitors are particularly effective in suppressing the innate immune response.

## Supplementary Information

Supplementary Material 1.

Supplementary Material 2.

Supplementary Material 3.

Supplementary Material 4.

Supplementary Material 5.

Supplementary Material 6.

Supplementary Material 7.

Supplementary Material 8.

Supplementary Material 9.

Supplementary Material 10.

Supplementary Material 11.

Supplementary Material 12.

Supplementary Material 13.

Supplementary Material 14.

## Data Availability

All data generated or analyzed during this study are included in this published article and its supplementary information files.

## Abbreviations

2DG: 2-deoxyglucose
3BP: 3-bromopyruvate
ADAM8: A disintegrin and metalloproteinase domain-containing protein-8
ADAMTS9: A disintegrin and metalloproteinase with thrombospondin motifs-9
AUC: Area under the curve
BP: Biological Pathway
CCL2: C-C Motif Chemokine Ligand 2
CCR5: C-C Motif Chemokine Receptor 5
CD11b: Cluster of Differentiation 11b (Integrin Subunit Alpha M protein)
CPM: Counts per million
CtBP: Carboxyl-terminal binding protein
DAM: Disease-activated microglia
DIM: Disease inflammatory macrophages
DSP: Digital spatial profiling
DMSO: Dimethyl sulfoxide
GFAP: Glial fibrillary acidic protein
GO: Gene ontology
GSVA: Gene Set Variance Analysis
HK1: Hexokinase-1
HK2: Hexokinase-2
Iba1: Ionized calcium-binding adaptor molecule-1 protein
IκBα: Inhibitor of κB alpha
IL1β: Interleukin-1 beta
i.p.: Intraperitoneal
LND: Lonidamine
NADH: Nicotinamide-adenine dinucleotide (reduced form)
NeuN: Neuronal Nuclei (RNA Binding Fox-1 Homolog 3 protein)
NFIA: Nuclear Factor I/A
NF-H: Neurofilament-H
NFκB: Nuclear factor kappa-B
PFA: Paraformaldehyde
Ribo.DAM2: Ribosomal disease-associated microglia-2
SOX9: SRY-Box Transcription Factor 9
S100B: S100 calcium-binding protein B
UMAP: Uniform manifold approximation and projection
γH2Ax: Phosphorylated H2A histone family member X
