# Supplementary material for: Transcriptional and neuroprotective effects of hexokinase-2 inhibitors administered after stroke
Source: J Neuroinflammation. 2025 Oct 28;22:247. doi: 10.1186/s12974-025-03594-1 (PMC12570821; doi:10.1186/s12974-025-03594-1)
Supplement: Supplementary file 14 — Supplementary Material 14. [file 12974_2025_3594_MOESM14_ESM.pdf]

Supplemental Table 1: Antibody sources and dilutions used

Antibody	Source	ID	Host	Dilution
Primary Antibodies				
Cofilin	Millipore, Temecula, CA, USA	51755	Rabbit	1:300
CD11b	Abcam Inc., Waltham, MA, USA	ab8878	Rat	1:200
γH2Ax	Cell Signaling, Danvers, MA, USA	9718S	Rabbit	1:500
Iba1	Wako, Richmond, VA, USA	019-19741	Rabbit	1:1000
NeuN	Millipore, Temecula, CA, USA	ABN78	Rabbit	1:1000
NeuN	Synaptic systems, Temecula, CA, USA	26604	Guinea Pig	1:500
Neurofilament-H	BioLegend, San Diego, CA, USA	801601	Mouse	1:500
Secondary Antibodies				
anti-rabbit IgG, Biotinylated	Vector Lab, Burlingame, CA, USA	BA-1000	Goat	1:250
anti-guinea pig IgG 594	Jackson Laboratory, Bar Harbor, ME, USA	106-585-003	Goat	1:1000
anti-guinea pig IgG 647	Thermo Fisher Scientific, Waltham, MA, USA	A21450	Goat	1:1000
anti-mouse IgG 488	Thermo Fisher Scientific, Waltham, MA, USA	A21202	Donkey	1:1000
anti-rabbit IgG 488	Thermo Fisher Scientific, Waltham, MA, USA	A21206	Donkey	1:1000
anti-rat IgG 594	Thermo Fisher Scientific, Waltham, MA, USA	A11007	Goat	1:1000

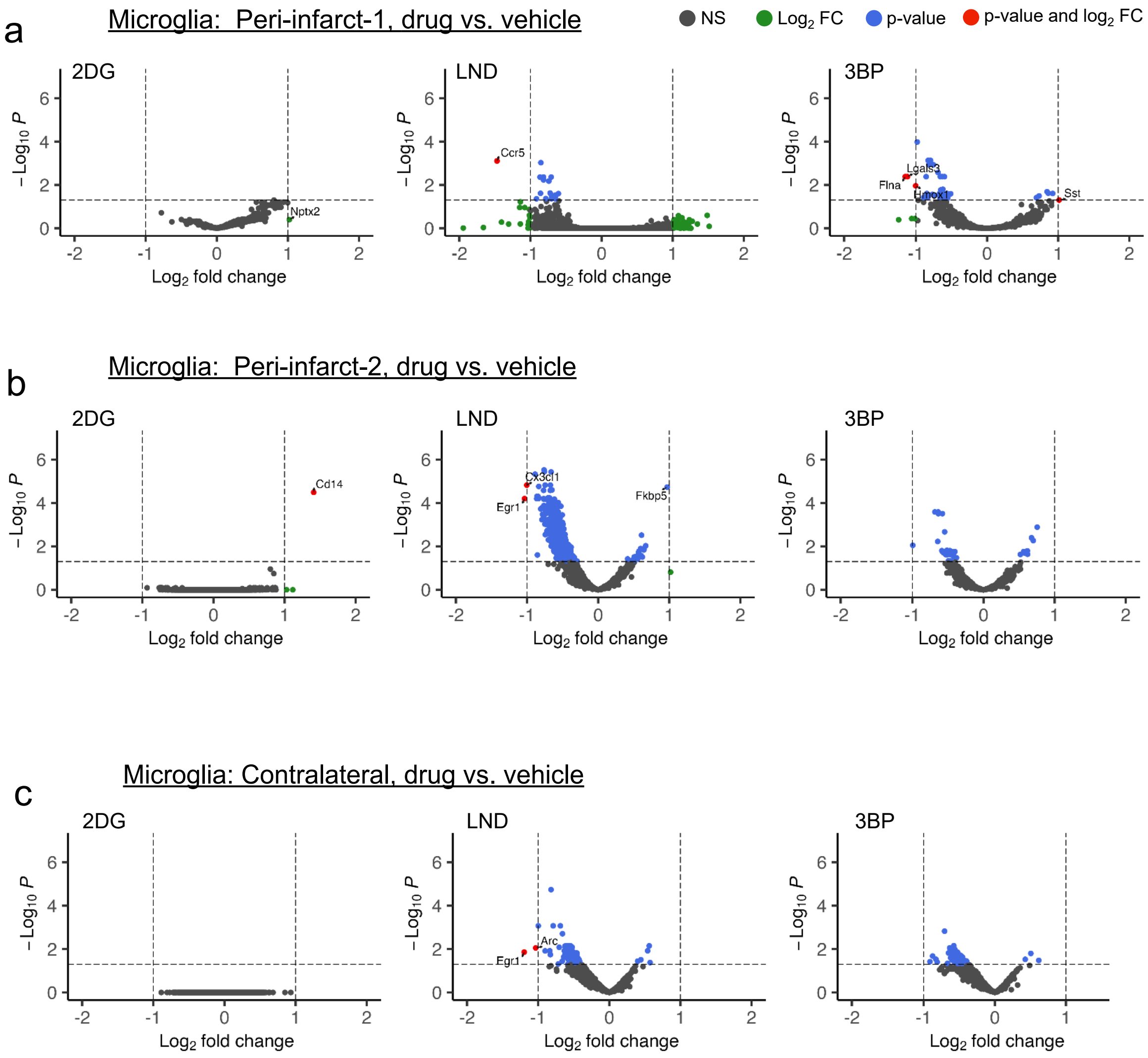

Supplemental Figure 1. **Microglial gene expression displayed as a comparison of each HK2 inhibitor vs. vehicle.** Data are from the (**a**) peri-infarct-1 cortex, (**b**) peri-infact-2 cortex, and (**c**) contralateral cortex. These data correspond to Fig. 2. Genes with log_2 expression fold change (FC) > 1 and p < 0.05 are denoted by red dots; log_2 FC > 1 and p > 0.05 by green dots; log_2 FC < 1 and p < 0.05 denoted by blue dots, and not significant (NS) by black dots. n = 4 mice under each of the 4 drug treatment conditions (n = 16 total).

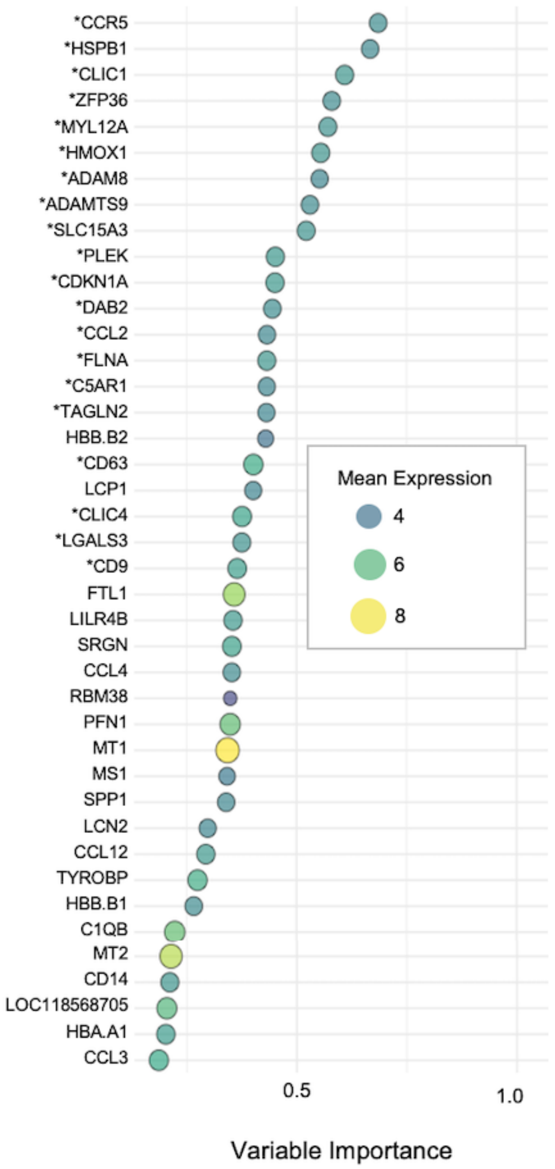

Supplemental Figure 2. Gene set defining ischemia-activated microglia. A random forest classification was used to identify a gene set in which expression changes are most characteristic of the changes observed in peri-infarct-1 microglia relative to contralateral microglia. Top scoring genes that were also highly expressed in microglia (denoted by *) comprised the activated microglial signature gene set displayed in Fig. 2. The size and color of the circles indicate the normalized gene expression levels.

a

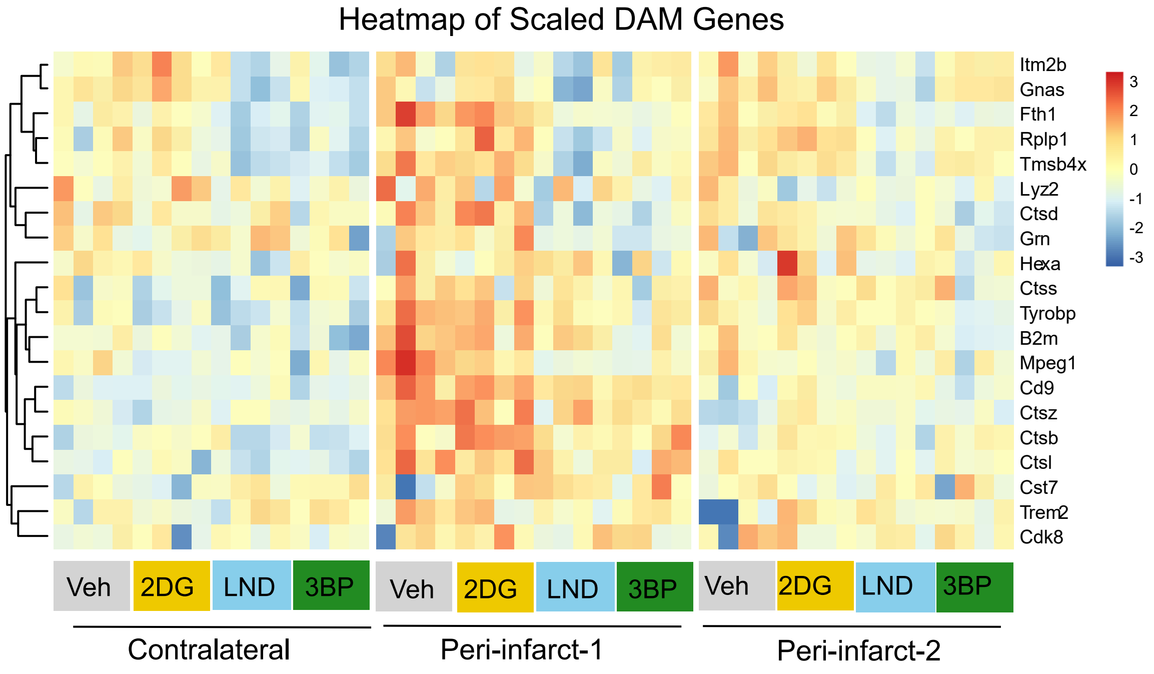

b

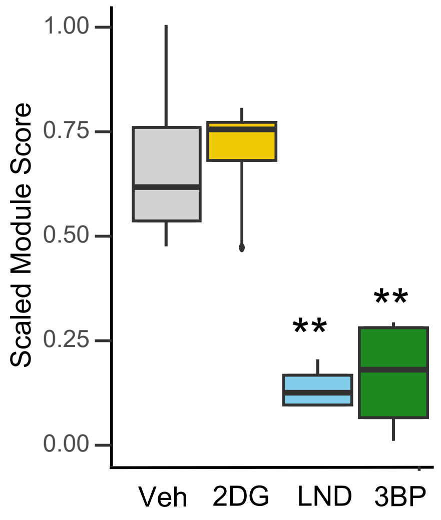

Supplemental Figure 3. Microglial expression of neurodegenerative disease-associated genes. a. Heatmap showing z-scaled expression of genes previously identified as upregulated in neurodegenerative disease-associated microglia (DAM) (Keren-Shaull et al., 2017). Data are shown for each treatment condition and region of interest; n = 4 for each treatment group. **b.** Box plot showing effects of drug treatment on the normalized composite expression of the upregulated DAM gene set in peri-infarct-1 microglia. ** $p < 0.01$ vs vehicle by ANOVA with Dunnett's test.

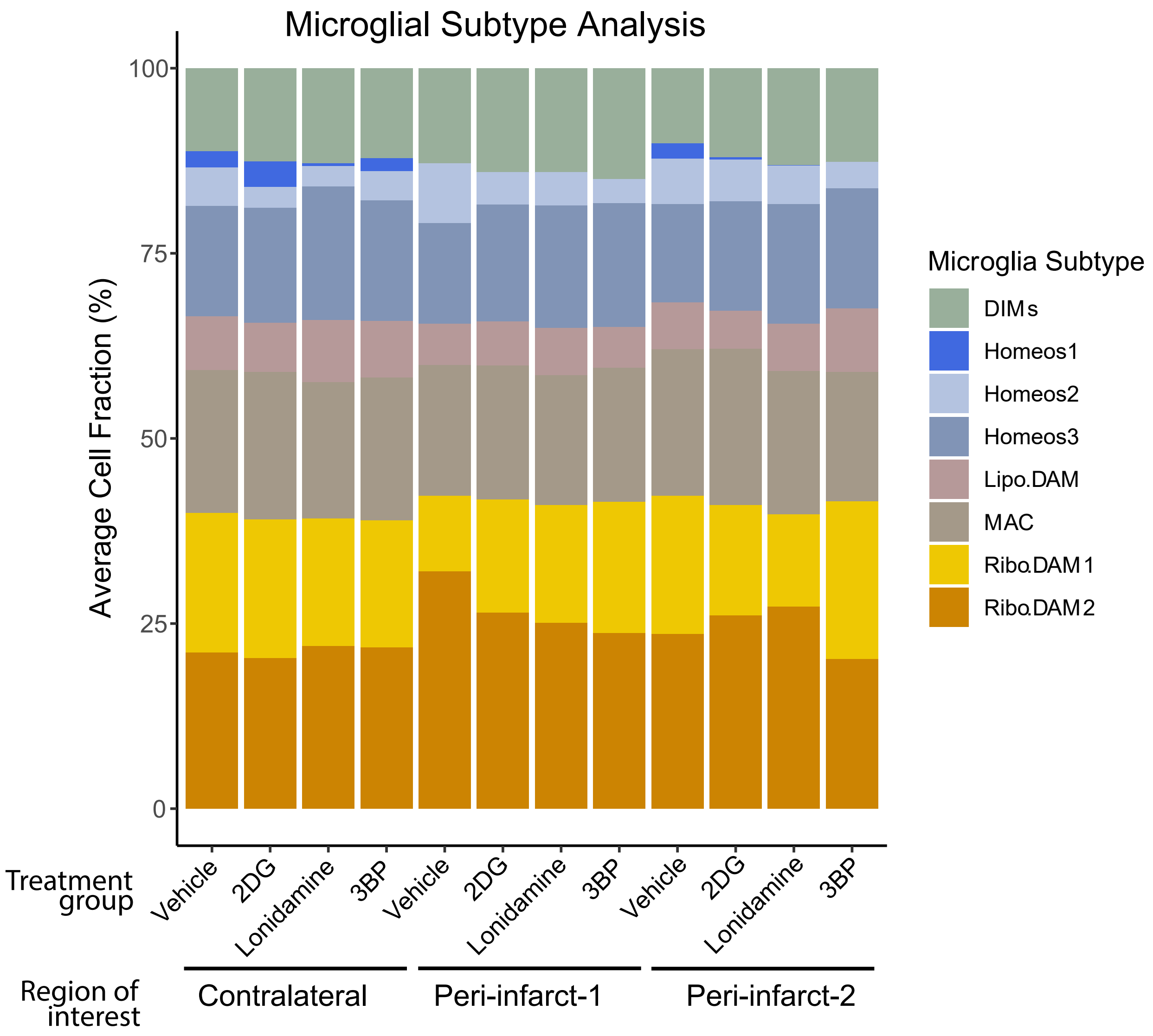

Supplemental Figure 4. CIBERSORTx analysis of microglial subtypes
ANOVA with Dunnett's test revealed significant spatial differences in Homeostatic-1 (Homeos1) microglia ($p = 0.005$), disease inflammatory macrophages (DIMs; $p < 0.001$), ribosomal disease-associated microglia-2 (Ribo.DAM2; $p = 0.002$), and border-associated macrophages (MAC; $p = 0.020$), comparing the peri-infarct-1 vs. contralateral regions. Significant drug treatment effects were observed for DIMs ($p = 0.004$) and Homeostatic-3 (Homeos3) microglia ($p = 0.010$), with 3BP and LND increasing the abundance of both subtypes. No significant spatial × treatment interactions were identified. n = 4. Lipo.DAM, Lipid droplet accumulating disease associated microglia.

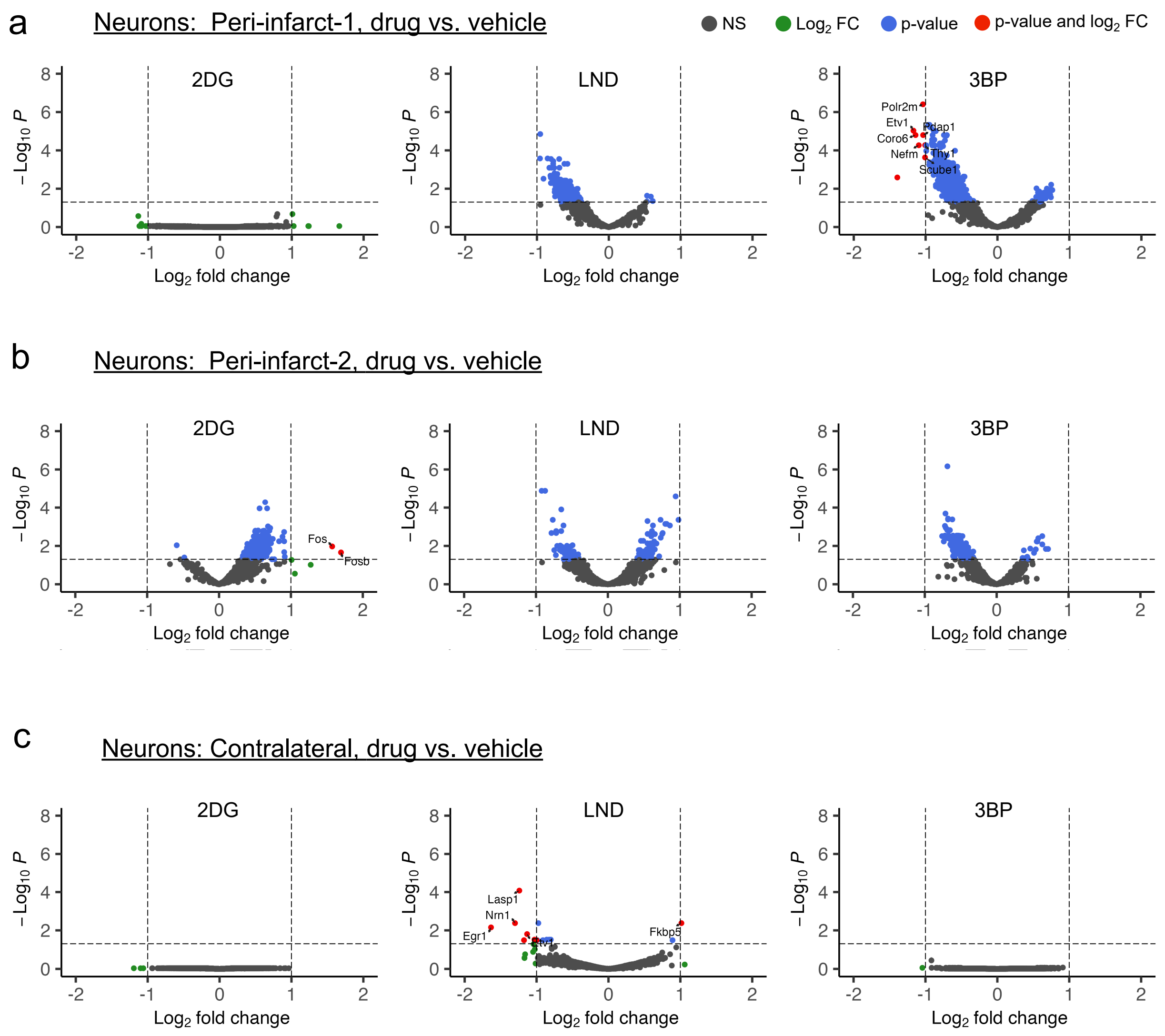

Supplemental Figure 5. Effects of HK2 inhibitors on peri-infarct neuronal gene expression
Data are from the (**a**) peri-infarct-1 cortex, (**b**) peri-infact-2 cortex, or (**c**) contralateral cortex. Genes with $\log_2$ expression fold change (FC) > 1 and p < 0.05 are denoted by red dots; $\log_2$ FC > 1 and p > 0.05 by green dots; $\log_2$ FC < 1 and p < 0.05 denoted by blue dots, and not significant (NS) by black dots. n = 4.

Immune Response

1. Natural killer cell activation
2. Regulation of macrophae activation
3. TNF superfamily cytokine production
4. Macrophage chemotaxis
5. Macrophage migration
6. Monocyte chemotaxis
7. Activation of immune response
8. Cytokine production

Metabolic

1. ATP metabolic processes
2. Glucose-6-phosphate metabolic processes
3. Glutamate metabolic processes
4. Glutathione metabolic processes
5. Cellular homeostasis
6. Oxidative phopsphorylation
7. Regulation of mitochondrial membrane permeability
8. Superoxide metabolic processes

Cell Stress/Death

1. Apoptotic mitochondrial changes
2. Cell death in response to oxidative stress
3. Apoptotic signaling pathway
4. Cellular response to ROS
5. Cellular response to unfolded proteins
6. Neuronal death
7. Neuronal death in response to oxidative stress
8. Endoplasmic reticulum unfolded protein response

Supplemental Figure 6. Gene functional pathways corresponding to Figure 3b.

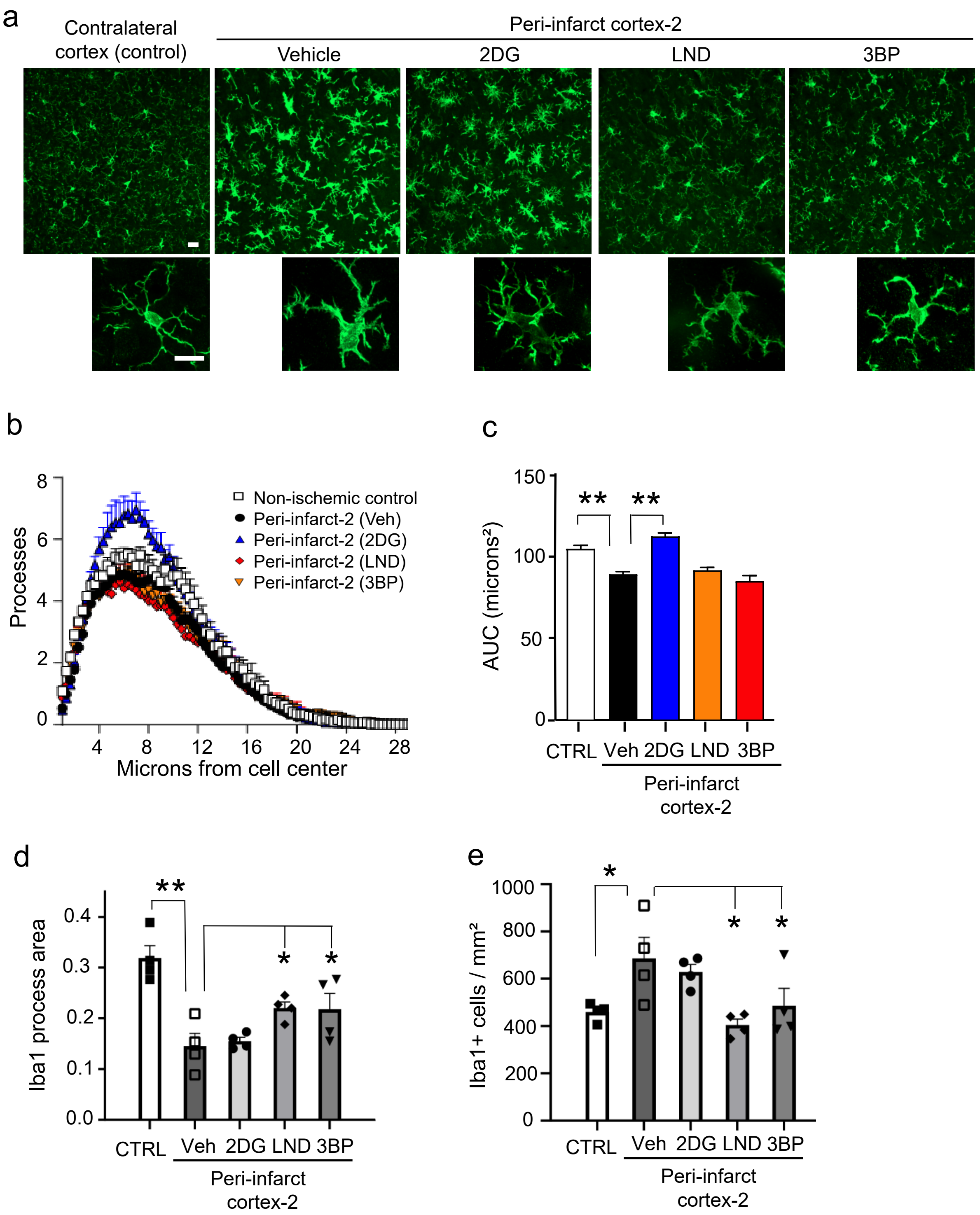

Supplemental Figure 7. Microglia morphology in peri-infarct-2 cortex
a. Microglial morphology as shown by Iba1 immunolabeling. High magnification images show morphologies representative of each treatment group. Scale bars = 10 μm. **b,c** Sholl analysis showing the mean number of microglial processes at distances from the cell center. This is quantified as area under the curve (AUC) . d. Mean microglial process area.
e. Microglia density. n = 4; * $p < 0.05$, ** $p < 0.01$ by ANOVA and Dunnett's test vs. vehicle.
